# Obstetrics: The Science and Art

**Published:** 1863

**Authors:** 


For sale by W. B. Keen & Co., Booksellers, Chicago.

---

OBSTETRICS: THE SCIENCE AND ART. By CHARLES D. MEIGS, M.D., lately Professor of Midwifery and the Diseases of Women and Children in Jefferson Medical College at Philadelphia, &c., &c., &c., &c.

Fourth edition revised, with 129 illustrations. PHILADELPHIA: BLANCHARD & LEA. 1863.

The high and well-earned reputation of Dr. MEIGS, together with the well-known character of his work on *obstetrics*, renders it unnecessary for us to enter upon any details in regard to the present edition. It is issued in the very best style of the publishing art; and is eminently worthy of the study of every student and practitioner of midwifery.

